# Surface engineering of graphitic carbon nitride polymers with cocatalysts for photocatalytic overall water splitting

**DOI:** 10.1039/c7sc01747b

**Published:** 2017-06-06

**Authors:** Guigang Zhang, Zhi-An Lan, Xinchen Wang

**Affiliations:** a State Key Laboratory of Photocatalysis on Energy and Environment , College of Chemistry , Fuzhou University , Fuzhou 350002 , China . Email: xcwang@fzu.edu.cn

## Abstract

Graphitic carbon nitride based polymers, being metal-free, accessible, environmentally benign and sustainable, have been widely investigated for artificial photosynthesis in recent years for the photocatalytic splitting of water to produce hydrogen fuel. However, the photocatalytic stoichiometric splitting of pure water into H_2_ and O_2_ with a molecular ratio of 2 : 1 is far from easy, and is usually hindered by the huge activation energy barrier and sluggish surface redox reaction kinetics. Herein, we provide a concise overview of cocatalyst modified graphitic carbon nitride based photocatalysts, with our main focus on the modulation of the water splitting redox reaction kinetics. We believe that a timely and concise review on this promising but challenging research topic will certainly be beneficial for general readers and researchers in order to better understand the property–activity relationship towards overall water splitting, which could also trigger the development of new organic architectures for photocatalytic overall water splitting through the rational control of surface chemistry.

## Abstract 2

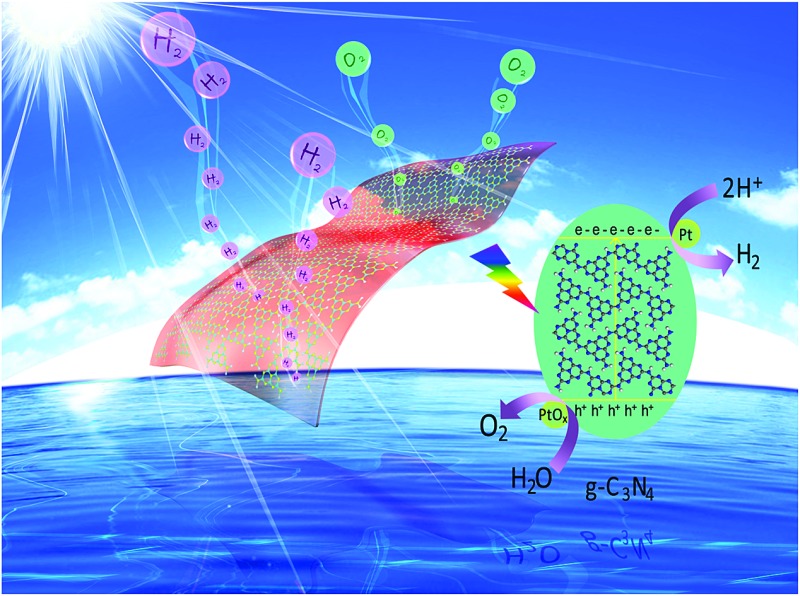
Overall water splitting for the stoichiometric generation of H_2_ and O_2_ has been achieved by rational cocatalyst modification of g-C_3_N_4_ polymers to modulate the surface redox reaction kinetics.

## 1. Introduction

Photocatalytic overall water splitting using nanoparticulate semiconductors is regarded as a potentially scalable and economically feasible method to convert the cost-free and earth-abundant solar energy into clean and renewable hydrogen fuel.^1–6^ Substantial research efforts have mainly focused on improving the photocatalytic activities of the semiconductors, and mostly focus on modification of the composition, structure, texture and morphology of the materials.^7–15^ However, until now, most of the materials that have been reported for water splitting reactions have been based on inorganic semiconductors,^16,17^ which are usually composed of rare elements and are not suitable for large-scale sustainable development. It is therefore reasonable to explore the use of earth-abundant materials, namely materials that are accessible, cost-affordable, and environmentally benign, and their textural and optical properties which can also be easily tailored to achieve photocatalytic water splitting with high performance.

In very recent years, organic conjugated polymers have attracted particular research interest since melon based carbon nitride polymers and crystalline graphitic carbon nitrides (both traditionally named as g-C_3_N_4_ for simplicity), which have been reported to be promising visible light photocatalysts, are accessible to many different researchers. They can be prepared in large amounts and can be used in studies on reactor and process design.^18–27^ Compared with traditional inorganic semiconductors, conjugated polymers possess more advantages, including being metal-free, nontoxic and low-cost, and the fact that their composition, structure and properties can also be readily tuned by adjusting the building blocks of versatile organic protocols. Furthermore, the organic texture of carbon nitride enables it to exhibit some unique properties, such as an abundance of nitrogen lone-pair electrons and grain boundary defects, thus making it very suitable for the construction of metal/carbon nitride heterojunctions for fast charge transfer at the interface. The first publication on carbon nitride photocatalysis has been cited >3000 times since 2009, as reported by Google Scholar. This has motivated extensive research into artificial photosynthesis using polymers, and a series of conjugated polymers have been investigated as excellent organic semiconductors for photocatalytic H_2_ production from water.^18–27^ In the presence of cocatalysts (*e.g.* Pt or Pd) and electron donors (*e.g.* triethanolamine or methanol), some of the conjugated polymers could achieve sufficient activities for visible light H_2_ production. Until now, the apparent quantum yield (AQY) of conjugated polymers for H_2_ evolution under visible light irradiation (*λ* > 420 nm) that could be obtained was as high as 38.8%.^28^ However, only a few of those investigated conjugated polymers have exhibited activity towards water oxidation, due to being restrained by their weak stability and insufficient oxidation ability of the valence holes. Therefore, it is observed that only a limited number of conjugated polymers can be utilized for overall water splitting.

By virtue of their well configured band structures (CB: –1.3 V; VB: 1.4 V *vs.* NHE, pH = 6.8) and robust stability, both of which are normally regarded as the two prerequisites for water splitting, melon-based g-C_3_N_4_ polymers have demonstrated promising capability for photocatalytic overall water splitting. In order to improve their photocatalytic activities, strategies such as doping,^29–34^ copolymerization,^35–39^ nanostructure engineering,^40–45^ hybridization^46–50^ and sol–gel^51^ modification have been developed to modify the composition, structure and optical properties of g-C_3_N_4_ polymers. It should be noted that pure g-C_3_N_4_ only absorbs visible light below *ca.* 470 nm; expansion of the optical absorption threshold through the use of strategies such as doping or copolymerization is therefore regarded as a feasible way to improve the photocatalytic efficiency from the viewpoint of enhanced energy input. However, in most cases, pure g-C_3_N_4_ without cocatalysts cannot exhibit sufficient photoactivity due to the huge activation energy and sluggish surface reaction kinetics. Thus, suitable H_2_ and O_2_ evolution cocatalysts, *i.e.* Pt,^52–55^ Rh,^56^ MoS_2_,^57–60^ and Co_3_O_4_,^61–63^ are indispensable to separately promote the H_2_ and O_2_ evolution reaction rate. On the one hand, cocatalysts could serve as charge carrier trap centres in order to quickly extract electrons or holes from the bulks of semiconductors to the interfaces of the cocatalysts. Because a Schottky barrier can be formed at the metal/g-C_3_N_4_ interface, noble metals with larger work functions, that is, a lower Fermi level, should trap electrons more readily. Thus Pt, among the many noble metals, with the largest work function, is the best cocatalyst for trapping electrons. Consequently, most of the light excited charge carriers would immediately participate in the subsequent surface redox reaction without recombination. On the other hand, a volcano relationship between the exchange current for H_2_ evolution and the M–H strength (M is a transition metal) was observed. Among the investigated transition metals, Pt was at the peak of the volcano, and showed the lowest activation energy for hydrogen evolution. It is thus desirable to imagine that the cocatalyst plays key roles in controlling the surface redox reaction kinetics, which are normally confirmed to represent the rate determining step of the overall water splitting process. Actually, the physicochemical properties of the cocatalysts, including their electron trapping ability, exchange current for H_2_ evolution, structure and morphology, are closely related to their photocatalytic activity.^64–67^ It is therefore of pivotal importance to investigate and conclude the property–activity relationships of H_2_ and O_2_ evolution cocatalysts.

Herein, we review the recent developments of g-C_3_N_4_ based polymers for the water splitting reaction, which mainly focus on cocatalyst modifications in order to tune the surface reaction kinetics and thus optimize the photocatalytic activities. We believe that a timely and systematic review into this very interesting and hot research topic will certainly be beneficial for general researchers to gather more experimental and theoretical details for a better understanding of the water splitting reaction mechanism. This will also open up new avenues to guide the development of new artificial photosynthesis systems by systematically configuring and engineering other promising organic architectures from the viewpoint of surface reaction kinetics modulation.

## 2. Fundamentals of water splitting

Photocatalytic overall water splitting for the stoichiometric generation of H_2_ and O_2_ in a molecular ratio of 2 : 1 is a thermodynamically uphill reaction that requires additional energy input, usually solar energy, to drive this multiple-electron-proton transfer and hugely energy consuming process. In principle, semiconductors that are theoretically capable of catalyzing the overall water splitting reaction are restricted:^68,69^ (i) the band gap energy should at least be larger than 1.23 eV (to meet the thermodynamic barrier, ideally the band gap should be 2.0 eV after considering the kinetic barriers);^70^ (ii) the conduction (valence) band potentials should be sufficiently negative (positive) for the water reduction (oxidation) half reactions; (iii) semiconductors should be robust enough towards light and solution corrosion.^71^ Except for these strictly defined requirements, polymers should also be well designed to capture sufficient visible photons for the excitation and generation of charge carriers (electrons and holes), which are involved in the subsequent water splitting reaction.^6^ In addition, conjugated polymers should exhibit excellent structural and textural properties, which are required for the fast separation and transfer of light-induced charge carriers to the interfaces of the photocatalysts without recombination. Therefore, it is certainly a great challenge to achieve overall water splitting through the rational construction of conjugated polymers.

**Fig. 1:**
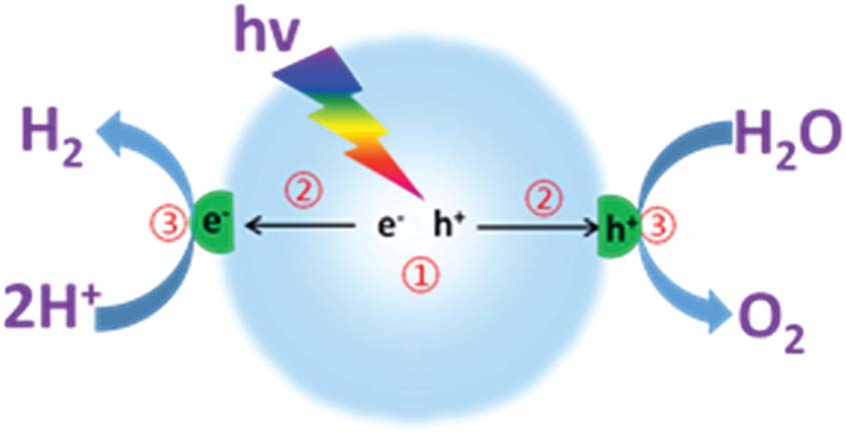
Schematic illustration of photocatalytic overall water splitting over a semiconductor photocatalyst modified with both H_2_- and O_2_-evolution cocatalysts.

As shown in Fig. 1, photocatalytic overall water splitting by a nanoparticulate photocatalyst generally involves three major steps: (i) semiconductors absorb incident light at an energy larger than the band gap for the excitation and generation of charge carriers; (ii) charge carrier separation, migration and transfer to the interfaces of semiconductors without recombination; (iii) electrons and holes separately react with the adsorbed protons and water for H_2_ and O_2_ evolution at the interface. It should be noted that the first two steps are closely related to the thermodynamic properties, such as optical properties, specific surface area, texture and microstructure of the semiconductors.^68^ Over the past few decades, substantial efforts have been devoted to developing promising semiconductors with enhanced optical absorption efficiency and optimized charge carrier behaviour.^52,55^ For instance, a variety of well-designed chemical strategies have been demonstrated to enhance the optical absorption, enlarge the surface area, and optimize the electronic and texture properties, thus improving the water splitting activities.^63^ However, the reaction rate of the third step is confirmed to be much slower than that of the first two steps, which normally involve the use of cocatalysts to decrease the reaction activation energy and accelerate the H–O bond breaking and O–O bond formation. Evidently, the last step is the major step for kinetic control of the overall water splitting efficiency. It is thus of pivotal importance to develop suitable cocatalysts to improve the water redox reaction efficiency and reinforce the stability of the semiconductors.

**Fig. 2:**
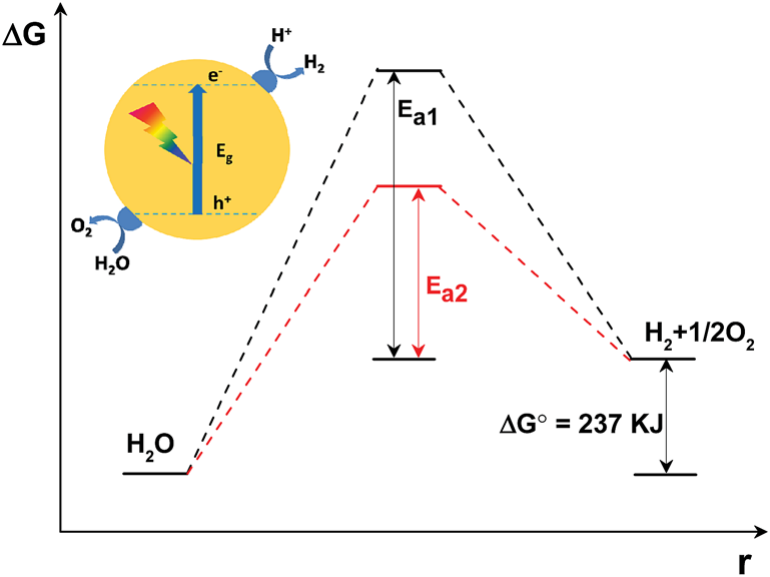
Schematic description of the energy diagram of a semiconductor modified with both H_2_ and O_2_ evolution cocatalysts under light irradiation for non-sacrificial photocatalytic water splitting. *E*_a1_: activation energy without cocatalysts; *E*_a2_: activation energy with cocatalysts; Δ*G*: Gibbs free energy change; Δ*G* ^0^: Gibbs free energy change under standard conditions; *r*: redox reaction process.

In principle, as is shown in Fig. 2, the Gibbs free energy change for the decomposition of 1 mol H_2_O to 1 mol H_2_ and 1/2 mol O_2_ under standard conditions is 237 kJ (corresponding to 1.23 eV). This reaction, despite only generating two singular molecules, is a thermodynamically uphill reaction that is non-spontaneous and usually calls for huge additional energy input (*e.g.* solar energy) to drive it.^3–5^ Furthermore, besides the additional energy input required, a huge energy barrier, *i.e.* the activation energy, usually hinders the water splitting reaction. The deposition of cocatalysts has been reported to promote photocatalytic activity.^64,65^ This is because, under light irradiation, the cocatalysts not only function as kinetic promoters to catalyse the evolution rate of the gases, but also serve as charge trap centres to extract electrons and holes from the photo-excited semiconductors. Evidently, the overall water splitting activity depends on the catalytic performance of the cocatalysts. In addition, in comparison with the water reduction half reaction, the water oxidation half reaction, which involves the transfer of four-electrons accompanied by O–H bond breaking and O–O bond formation, is usually restrained by the huge activation energy (∼700 mV) and sluggish O–O bond formation kinetics.^69^ Thus, the water oxidation process is usually more restrained in comparison with the water reduction half reaction and is thus regarded as the key step to achieving an efficient overall water splitting reaction.

In order to achieve overall water splitting, it is advisable to individually investigate the water reduction and water oxidation half reactions at first. After careful examination of the property–activity relationships of the cocatalysts in sacrificial H_2_ or O_2_ evolution reactions, it is thus reasonable to carry out an overall water splitting investigation. It should be noted that the factors dominating the quantum efficiency should not be identical for non-sacrificial water splitting accompanied by a large increase in the Gibbs energy and sacrificial hydrogen or oxygen evolution reactions. For instance, the loading technology for cocatalysts may make different contributions to the overall water splitting and sacrificial hydrogen or oxygen evolution reactions. Furthermore, the reverse reaction is always considered to be a critical issue in an overall water splitting system, while it is not taken into consideration in sacrificial H_2_ or O_2_ evolution systems. Nevertheless, we are able to learn of some key factors and useful experience from the sacrificial half reactions. Such knowledge is very helpful for investigations into overall water splitting. Following this line of inquiry, in this perspective we firstly investigate the water reduction and water oxidation reactions, mainly focusing on the control of the surface kinetics of g-C_3_N_4_ polymers. After that, we demonstrate how to achieve overall water splitting by careful surface modification of g-C_3_N_4_ polymers with well-designed H_2_ and O_2_ evolution promoters.

## 3. Water reduction half reaction

**Fig. 3:**
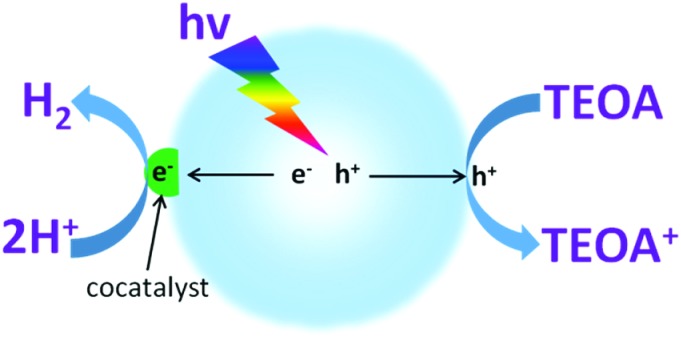
Schematic illustration of photocatalytic water reduction for H_2_ evolution in the presence of an electron donor driven by a semiconductor modified with H_2_ evolution cocatalysts. TEOA: triethanolamine.

To investigate the water reduction half reaction, electron donors (such as TEOA, methanol, ethanol, *etc.*) are usually added into the system to accelerate the hole oxidation process (Fig. 3).^52,55,72^ In most cases, the semiconductors themselves can only act as light transducers to absorb incident photons for the excitation and generation of charge carriers. Most of them require an additional catalyst, namely a cocatalyst, to accelerate the surface reaction kinetics and promote photocatalytic activity.^64,65^ In principle, cocatalysts could extract electrons from the interfaces of semiconductors, thus prolonging the charge carrier lifetime and restraining charge carrier recombination, both of which are largely beneficial for promoting the photocatalytic activity. Furthermore, noble metals, owing to their large work functions for trapping electrons and lowest activation energy for H_2_ evolution, are promising candidates for the catalysis of photocatalytic water splitting.^64^ Aside from their good catalytic properties, the cocatalysts could also suppress photo-corrosion and increase the stability of the semiconductor photocatalysts.

Many factors could affect the capability of H_2_ evolution cocatalysts to promote the photocatalytic water reduction reaction, such as the composition, loading contents, particle size, morphology and structure of the cocatalysts.^50–54^ Traditional cocatalysts that are suitable for promoting H_2_ evolution activity are mainly based on noble metals such as Pt,^50–54^ Pd,^73^ Rh,^56^ Au^74^ and Ag.^75,76^ Noble metal cocatalysts usually exhibit very low overpotentials, even being close to zero for the H_2_ evolution reaction, and thus demonstrate excellent performance for increasing the H_2_ evolution activity. However, their high cost, toxicity and scarcity restrict their applications when scaling up. Hence, it is urgent to develop alternative cocatalysts that use earth-abundant elements for sustainable solar energy conversion. Recently, some transition metals (NiO,^77^ MoS_2_,^57–60^ WS_2_,^78,79^ Ni(OH)_2_^80–82^ and CoP^83^) have also demonstrated an excellent ability to promote photocatalytic H_2_ evolution activity. Compared with noble metals, transition metals possess more advantages, such as their abundance, low-cost and low toxicity, thus making them suitable candidates for sustainable applications. Furthermore, other types of cocatalysts, such as graphene and carbon quantum dots,^48,84^ can also serve as versatile cocatalysts to promote H_2_ evolution activity. In this section, we will discuss the effects of different cocatalysts on the promotion of H_2_ evolution activity, with the aim to better understand their property–activity relationships.

### 3.1 Noble metal cocatalysts

**Fig. 4:**
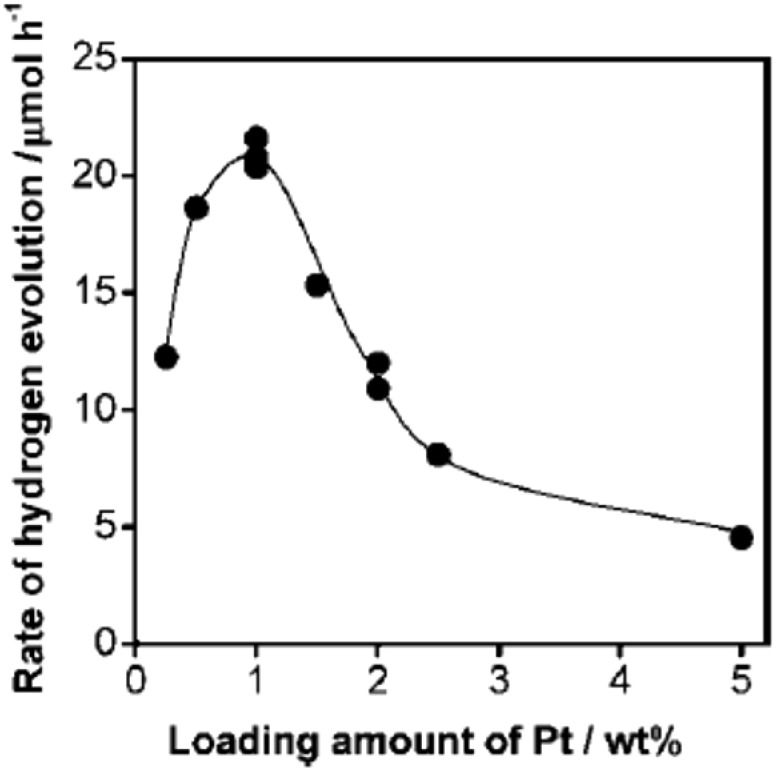
Dependence of the H_2_ evolution rate, when using Pt-loaded g-C_3_N_4_ under visible light, on the loading amount of Pt. Reprinted with permission from ref. 56. Copyright 2009, American Chemical Society.

As was discussed above, many different noble metals have been investigated as effective cocatalysts to promote photocatalytic H_2_ evolution activity. However, Pt proves to be the most effective cocatalyst for H_2_ evolution due to its largest work function for trapping electrons and its lowest activation energy for H_2_ evolution. For instance, when a Ru nanoparticle was deposited on g-C_3_N_4_ polymers as a H_2_ evolution cocatalyst, the visible light H_2_ evolution rate that was achieved was 2.1 μmol h^–1^.^56^ On the contrary, when the same amount of Pt cocatalyst was loaded onto the surface of pure g-C_3_N_4_, the visible light H_2_ evolution rate was increased to as high as 7.3 μmol h^–1^, which was 3.5 times higher than that of the Ru cocatalyst, thus indicating the great advantage of Pt in promoting H_2_ evolution activity.^56^ Furthermore, the loading contents of the H_2_ evolution cocatalysts could also largely affect the photocatalytic activity (Fig. 4). At lower loading contents, the activity increases when adding higher amounts of cocatalyst, which is mainly due to there being more active sites available for redox reactions. However, when excessive amounts of cocatalyst were deposited on the surfaces of the semiconductors, fewer surface active sites were exposed to absorb the reactive species. Furthermore, excessive cocatalyst amounts on the surfaces of semiconductors will shield the incident light and prevent light absorption. In addition, excessive cocatalyst amounts would stimulate particle aggregation which is certainly bad for the activity of the cocatalyst. Finally, high loading amounts of cocatalyst may also function as the charge carrier recombination centre and thus shorten the lifetime of the electron–hole pairs.

In general, noble metals are usually prepared by the reasonable reduction of metal precursors (*e.g.*, H_2_PtCl_6_) from a liquid solution.^29^ Depending on the reduction methodology used, the as-prepared cocatalysts always exhibit quite different abilities for the promotion of the photocatalytic H_2_ evolution reaction. Up until now, the most popular method for obtaining noble metal cocatalysts has been through *in situ* photo-deposition from aqueous noble metal precursors, due to the facile fabrication process and highly efficient activities. For instance, when a certain amount of H_2_PtCl_6_ aqueous solution was added into the photocatalytic reaction system, homogeneous Pt species with particle sizes of about 3–5 nm were deposited on the surface of carbon nitride.^56^ In this case, the semiconductor absorbs light to generate excited electrons and holes. The adsorbed Pt^6+^ is reduced to metallic Pt by light excited electrons and is subsequently deposited *in situ* on the surface of carbon nitride. These metallic Pt particles could act as efficient cocatalysts to extract electrons from the bulk and reduce the H_2_ evolution over-potential. Compared to carbon nitride without modification by a Pt cocatalyst, the as-prepared Pt–g-C_3_N_4_ samples exhibited greatly enhanced activity, increased by a factor of 7, towards H_2_ evolution, thus reflecting the fact that Pt is an excellent cocatalyst for promoting H_2_ evolution activity.^56^

**Fig. 5:**
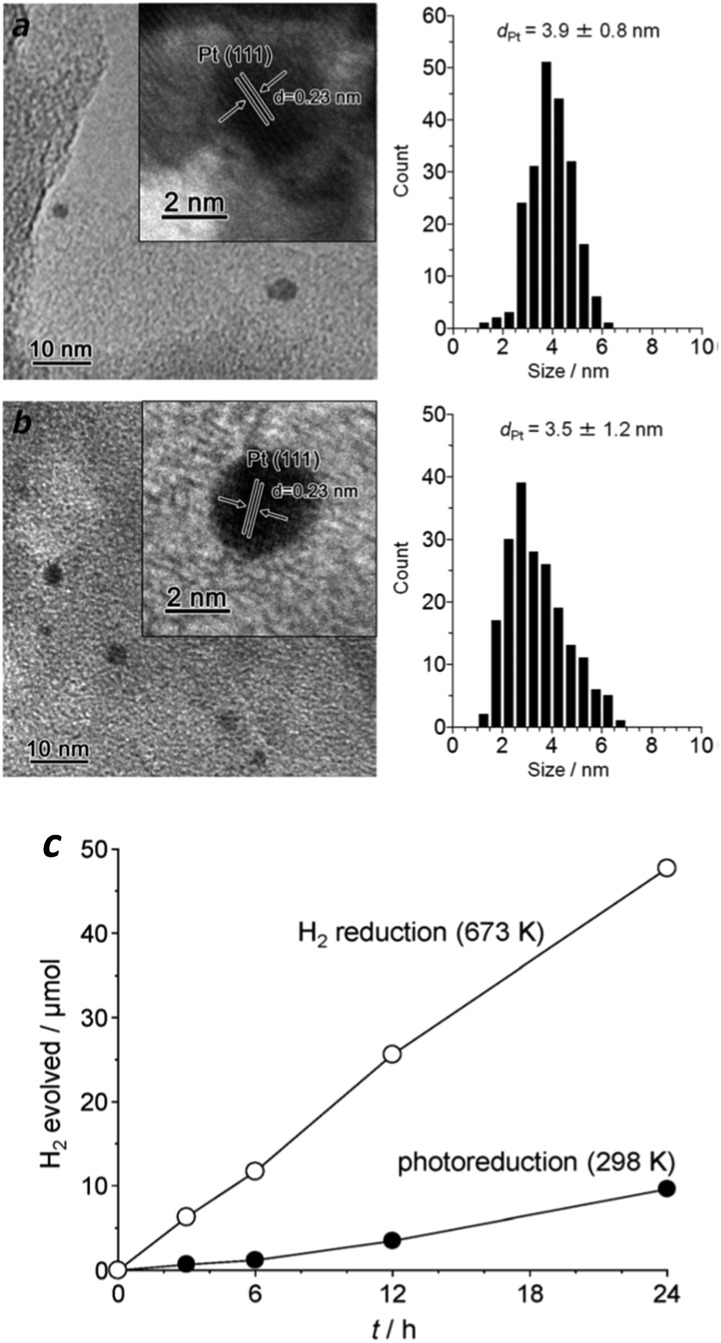
Size distribution of Pt particles prepared by (a) photoreduction (298 K) and (b) H_2_ reduction (673 K); (c) H_2_ evolution activities in the presence of an electron donor. Reprinted with permission from ref. 86. Copyright 2014, Royal Society of Chemistry.

Another popular method for noble metal cocatalyst deposition consists of impregnation followed by subsequent reduction under hot H_2_ flow. The particle size of metallic Pt prepared by this H_2_ reduction is also ultrafine, with the particles homogeneously deposited on the surface of g-C_3_N_4_ polymers, which is a similar outcome to that prepared by *in situ* photo-reduction (Fig. 5a and b), thus enabling it to be an active cocatalyst for the promotion of H_2_ evolution. Furthermore, the heating treatment during H_2_ reduction creates tight contact between the Pt nanoparticles and the carbon nitride polymers, which obviously favours the interface charge carrier transfer and improves the H_2_ evolution activity. As is shown in Fig. 5c, the Pt nanoparticles prepared by H_2_ reduction exhibited much higher H_2_ evolution activity than that of the Pt nanoparticles obtained by *in situ* photoreduction. Furthermore, the results also demonstrate good stability over the long reaction time, thus demonstrating it is promising for the promotion of photocatalytic H_2_ production activity. However, the photoreduction strategy was always used in previous reports for examination of the activities of the g-C_3_N_4_ polymers due to the facile but effective operation process. Until now, by modifying the properties and surface reaction kinetics, the highest H_2_ evolution apparent quantum yield (AQY) of the g-C_3_N_4_ based polymers that can be achieved is as high as 50.7% (ref. 28) at 405 nm and 38.8% (ref. 28) at 420 nm, while Pt nanoparticles are deposited *in situ* as H_2_ evolution cocatalysts in the presence of TEOA as the electron donor.

Noble metals do however usually have some limitations, such as high cost, toxicity and scarcity, which largely restrict their applications when scaling up. Therefore, further investigations are desired to develop new materials that use sustainable, low-cost and environmentally benign components to promote the H_2_ evolution activity of g-C_3_N_4_ polymers.

### 3.2 Transition metal based cocatalysts

Transition metals (including Fe^3+^, Co^2+^ and Ni^2+^) always demonstrate promising properties for heterogeneous catalysis due to their versatile chemical states and good stability towards solution corrosion. These transition metals can also act as kinetic promoters to improve the catalytic activity. Recently, some transition metal compounds, such as NiO,^77^ MoS_2_,^57–60^ Ni(OH)_2_,^80–82^ CoP,^83^ NiS_*x*_ (ref. 87 and 88) and WS_2_,^78,79^ have been reported to exhibit excellent activities for the photocatalytic H_2_ evolution reaction. Depending on the composition, morphology and properties of the metal composites, they can also exhibit comparable activities to those of noble metals, thus demonstrating that they are promising candidates for a wide range of applications. In addition, compared with traditional noble metals, these transition metal complexes possess many advantages, such as low-cost, low-toxicity, abundant resources and a variety of properties. In this section, we will discuss the positive roles of different transition metals in the promotion of the photocatalytic H_2_ evolution reaction, with the major purpose of developing efficient and sustainable H_2_ evolution cocatalysts.

#### 3.2.1 Transition metal dichalcogenide (TMD) based cocatalysts

**Fig. 6:**
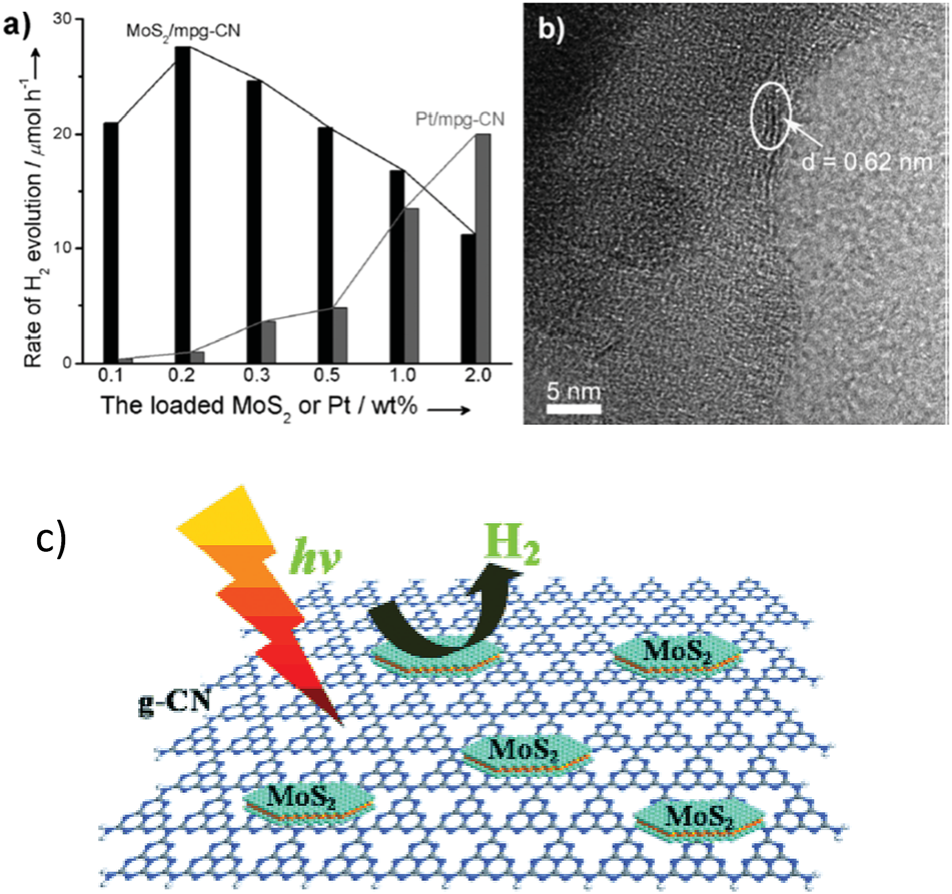
(a) The rate of H_2_ production over mpg-C_3_N_4_ loaded with different amounts of MoS_2_ or Pt, (b) TEM image of MoS_2_/g-C_3_N_4_ and (c) illustration of the deposition of layered MoS_2_ on the surface of g-C_3_N_4_ for photocatalytic H_2_ evolution. Reprinted with permission from ref. 57. Copyright 2013, Wiley-CVH.

The most popular noble metal-free cocatalysts are based on transition metal dichalcogenides (TMDs), such as MoS_2_ and WS_2_. Layered TMDs are easily fabricated and usually exhibit comparable electronic properties to those of graphene, thus making them promising candidates for electronic applications.^89^ These TMDs always exhibit narrow band gaps (usually lower than 1 eV), which present favourable optical properties, and thus they can be potentially used in photocatalytic applications. For instance, when layered MoS_2_ was deposited on the surface of g-C_3_N_4_, it greatly improved the photocatalytic H_2_ evolution activity.^57^ As is shown in Fig. 6, when the MoS_2_ loading content was below 1.0 wt%, the H_2_ evolution activities were higher than those of the Pt modified catalyst. This excellent activity may be ascribed to the similar layered geometries of MoS_2_ and graphitic g-C_3_N_4_, which are beneficial for facilitating interface charge carrier transfer and for prolonging the charge carrier life time. After modification with MoS_2_, the H_2_ evolution overpotential was dramatically decreased, which could promote the H_2_ evolution activity. Some other TMD based cocatalysts, *e.g.*WS_2_,^78,79^ NiS_*x*_ (ref. 87 and 88) and CoS_2_,^90,91^ could also serve as H_2_ evolution cocatalysts to promote the photocatalytic water reduction activity of g-C_3_N_4_ polymers. These successful investigations provide more opportunities for the design of sustainable H_2_ evolution catalysts. However, the stability and recyclability of these sulphides are a great concern. Future research is focused on advancing the stability of these materials, thus enabling their applications for scaling up.

### 3.2.2 Transition metal oxides or hydroxide based cocatalysts

Compared to metal sulfides, metal oxides exhibit much better stability towards solution corrosion. For instance, when a Ni@NiO core@shell nanostructure was constructed on the surface of g-C_3_N_4_ polymers, it demonstrated robust activity over a long reaction time.^77^ The NiO shell could act as a protective layer to prevent the size accumulation of the Ni core. Furthermore, only protons could penetrate the NiO shell and adsorb on the surface of the Ni core for H_2_ evolution. Other large sized species, such as O_2_, could not penetrate the shell and thus the backward reaction is avoided. Evidently, this could largely decrease the H_2_ evolution activation energy and promote the H_2_ evolution activity.

**Fig. 7:**
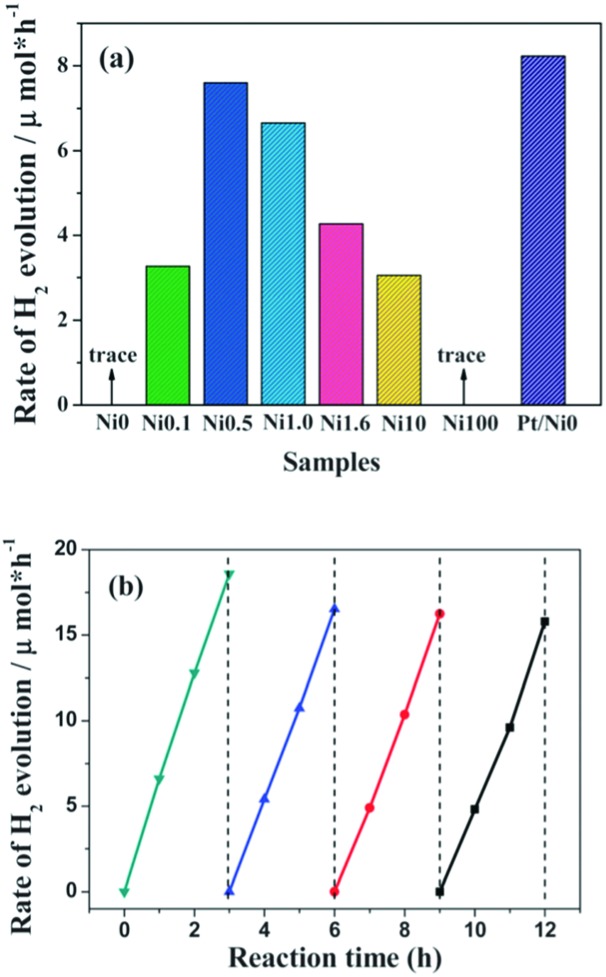
(a) Comparison of the photocatalytic activity of samples with different amounts of Ni(OH)_2_ loaded on g-C_3_N_4_ polymers, (b) cyclic H_2_-evolution curve for the Ni0.5 sample. Reprinted with permission from ref. 80. Copyright 2013, Royal Society of Chemistry.

Other than metal oxides, layered hydroxides, such as Ni(OH)_2_,^80–82^ are also excellent H_2_ evolution cocatalysts. For instance, Ni(OH)_2_ can function as a H_2_ evolution cocatalyst to combine with g-C_3_N_4_ for photocatalytic H_2_ production in the presence of an electron donor.^80^ As is shown in Fig. 7a, when Ni(OH)_2_ was deposited on g-C_3_N_4_ the binary composite demonstrated clearly enhanced H_2_ evolution activities in comparison with pure g-C_3_N_4_ without any cocatalyst. An adaptive interface developed between g-C_3_N_4_ and Ni(OH)_2_; this could promote interface charge carrier transfer and decrease recombination. Evidently, this contributes to promoting H_2_ evolution activity. Furthermore, the H_2_ evolution activities of the binary catalysts are closely related to the Ni(OH)_2_ loading amounts. Optimum activity was obtained when the loading percentage of Ni(OH)_2_ was 0.5 wt%. Meanwhile, the catalyst demonstrated comparable activities to that of the noble-metal Pt modified one. Furthermore, these Ni(OH)_2_ modified polymers also exhibit robust stability towards light and solution corrosion, which is necessary for practical applications. It is thus advisable to develop a noble-metal free system using all earth abundant elements for sustainable H_2_ production from water. Further improvements in the photocatalytic activity could potentially be obtained by reasonable adjustments of the composition, structure, morphology and properties of the transition-metal cocatalysts.

### 3.2.3 Artificial molecular based cocatalysts

Recently, some metal (Co, Ni and Fe) molecular systems have been developed which mimic the active sites of natural hydrogenases in plants, with high catalytic efficiencies for the reversible reduction of protons to molecular hydrogen.^92,93^ Being H_2_ active catalysts, hydrogenases possess unique properties, such as low activation energies for H_2_ evolution and a wide range of O_2_ sensitivities, while using organometallic catalytic sites composed of earth-abundant elements (*e.g.* Fe, Ni, S, C, N and O). However, one of the general problems of the currently used molecular systems is their instability upon long-term irradiation due to the existence of solution sensitive chemical bonds, *i.e.* the S–S bond. To overcome this issue, semiconductors with suitable band structures and robust stabilities are desired, both of which are believed to improve their efficiency and recyclability over long reaction times.

**Fig. 8:**
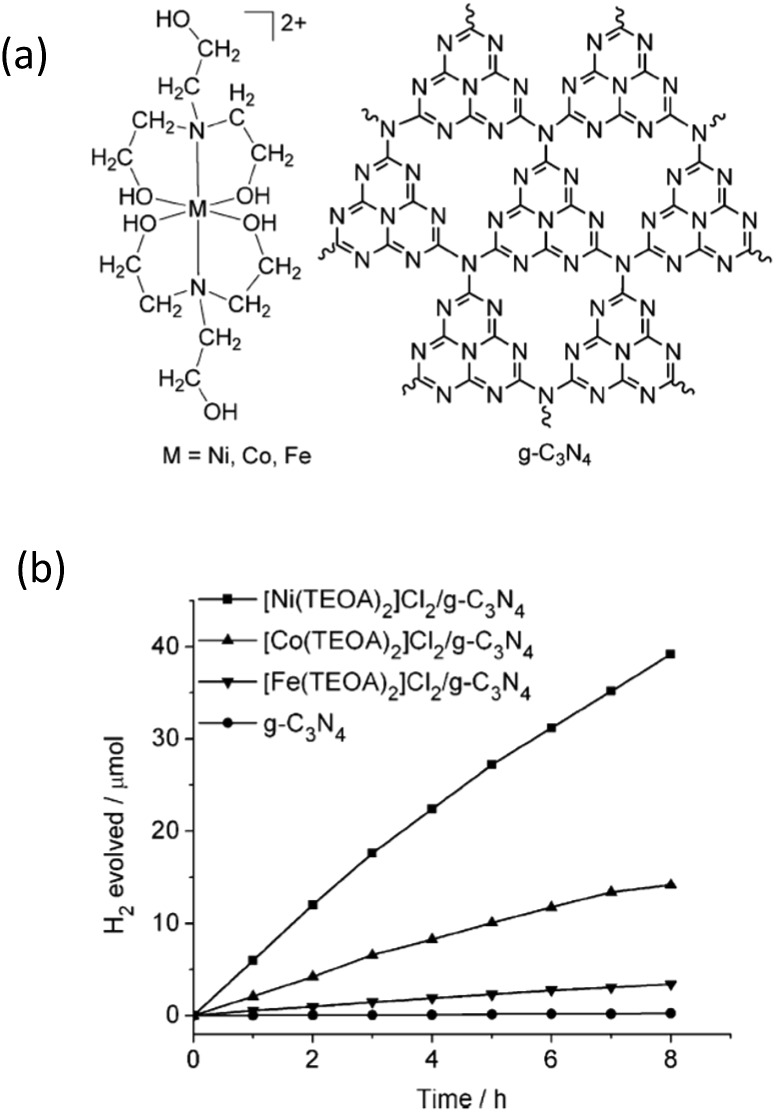
(a) Structures of Ni-, Co-, and Fe-based cocatalysts and g-C_3_N_4_, (b) photocatalytic H_2_-evolution for different cocatalyst modified g-C_3_N_4_ samples. Reprinted with permission from ref. 94. Copyright 2012, Wiley-CVH.

As a prototypical example, Sun *et al.* have developed a series of Co-, Ni- and Fe-based molecular systems to combine with g-C_3_N_4_ for photocatalytic H_2_ evolution.^94^ The molecular structure of the cocatalysts and their H_2_ evolution activities are shown in Fig. 8a and b. It can be seen in the figure that the Ni-based system demonstrates the best activity in comparison with the activities of the Co- and Fe-based catalysts for visible light H_2_ evolution, while pure g-C_3_N_4_ without any cocatalyst only generates trace amounts of H_2_. After adding 4 wt% of acriflavine as a photosensitizer to enhance the visible light absorption, the H_2_ evolution rate further increased and the amount of the evolved H_2_ gas reached 72 μmol over 8 h of irradiation, which corresponds to a TON of 106 based on Ni. This molecular catalytic system has been proven to be active towards photocatalytic H_2_ evolution over more than 60 h in aqueous solution, which is much longer than that of the previously reported molecular catalytic system with organic dyes or metal-containing complexes. Another water-soluble and functional synthetic hydrogenase, [Ni^II^(PPh_2_{NPhCH_2_P(O)(OH)_2_}_2_)_2_]Br_2_ (labelled as NiP for simplification), has been developed for use as a H_2_ evolution cocatalyst to combine with g-C_3_N_4_ for H_2_ production.^95^ The entirely synthetic g-C_3_N_4_–NiP system displays an unprecedentedly high TOF (109 h^–1^) and TON (166) for a hybrid system made of a molecular cocatalyst with g-C_3_N_4_ in aqueous solution. This synthetic g-C_3_N_4_–NiP system was active for 3 h of light irradiation. It is very interesting to observe that this system contains all sustainable elements (C, N, P and Ni) and no noble metals and organic solutions are used as solvents or electron donors. Obviously, this represents a green strategy for sustainable H_2_ production from the abundant resource of water.

However, up to now, most of the current examined molecular systems have only demonstrated limited lifetimes for reactions with long times, and are therefore not suitable for practical applications. Furthermore, the H_2_ production activities are still at a relatively low level. This main issue can be attributed to the poor interface charge carrier transfer between the water soluble molecular catalytic system and the insoluble solid state light transducers. Further investigations are required to facilitate the interface charge carrier transfer and prolong the lifetime of the molecular catalytic system in order to improve the photocatalytic H_2_ evolution activity.

### 3.3 Carbon based composite cocatalysts

**Fig. 9:**
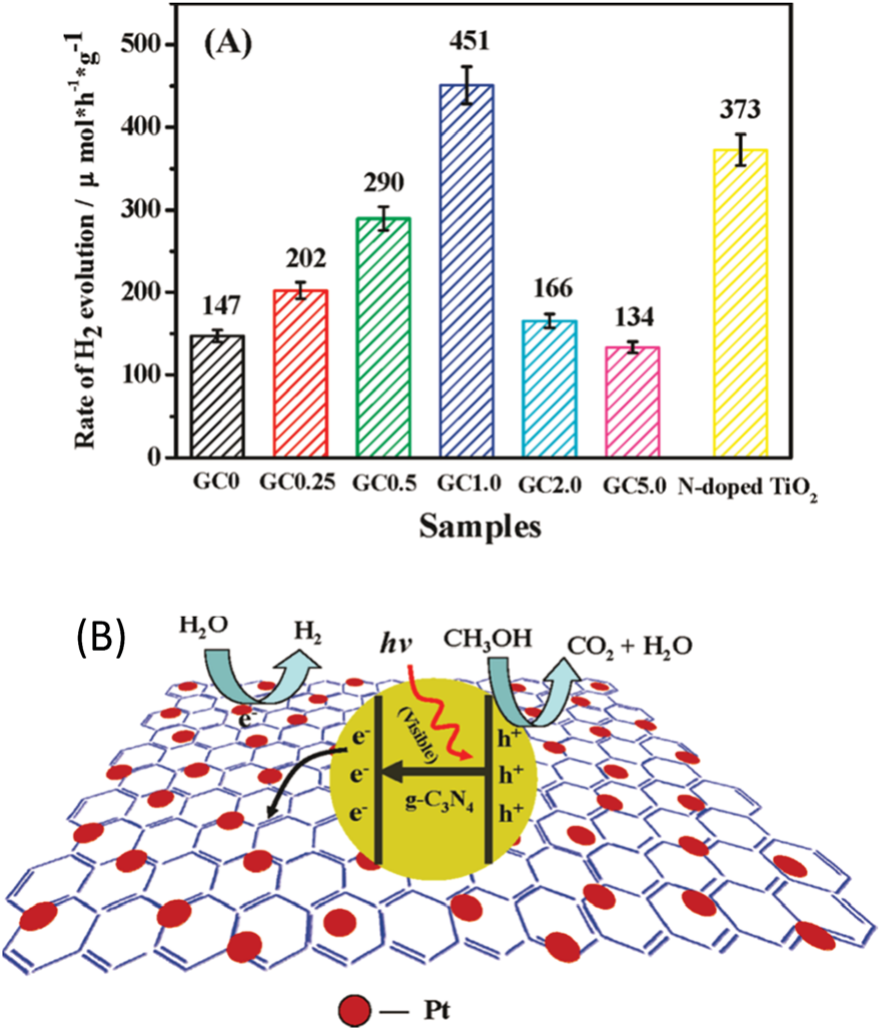
(A) Comparison of the photocatalytic activities of samples with different loading amounts of graphene on g-C_3_N_4_ polymers, (B) proposed mechanism for the enhanced electron transfer process of the graphene/g-C_3_N_4_ polymers. Reprinted with permission from ref. 48. Copyright 2011, American Chemical Society.

Carbon-based materials, such as 0D carbon dots,^84^ 1D carbon nanotubes (CNTs),^85^ and 2D nanosheets,^48^ always exhibit excellent electronic properties, thus making them promising candidates for electronic applications. Recently, these carbon-based materials have also found wide applications in the field of photocatalysis.^48,85,96^ This is because carbon-based materials can exhibit comparable conductivity to that of conductors, thus enabling them to act as an electron transport “highway” in order to accelerate charge carrier transfer and prolong the charge carrier lifetime. Evidently, these play determining roles in promoting the photocatalytic H_2_ evolution activity. Furthermore, carbon-based materials themselves can serve as H_2_ evolution cocatalysts or couple with other H_2_ evolution cocatalysts to further improve the photocatalytic H_2_ production activity. For instance, 2D graphene nanosheets incorporated with g-C_3_N_4_ polymers can greatly decrease the charge carrier recombination rate.^48^ Thus, more excited electrons are provided at the surfaces of the H_2_ evolution cocatalysts, which can be used for the proton reduction reaction. The optimum activity for photocatalytic H_2_ evolution was achieved when the deposition amount of graphene was determined as 1 wt%, and exceeded that of pure g-C_3_N_4_ by more than 3.07 times (Fig. 9a). The incorporated graphene nanosheet could quickly transfer excited electrons from the light transducer to the surface of the cocatalyst. In this case, the charge carrier recombination process is greatly decreased, which is certainly beneficial for the subsequent water redox reactions.

Very recently, Qu *et al.* developed a metal-free 3D graphitic carbon nitride/nitrogen-rich carbon nanofiber composite photocatalyst by *in situ* freeze drying fabrication.^85^ The open 3D system enlarges the specific surface area and provides more active cites for the surface H_2_ evolution reaction. On the one hand, the nitrogen-rich carbon nanofibers could function as charge carrier transfer mediators to accelerate the charge carrier mobility. On the other hand, they could also act as a H_2_ evolution cocatalyst to reduce the H_2_ evolution overpotential and facilitate the reduction kinetics of surface protons. When the composite photocatalyst was tested for photocatalytic H_2_ evolution in the presence of TEOA as the electron donor, the visible light H_2_ evolution activity of the 3D nanocomposites reached as high as 168.85 μ mol h^–1^, which was 18.3 times higher than that of the pure g-C_3_N_4_ polymer. It should be noted that no other noble metal materials are deposited as H_2_ evolution cocatalysts, thus revealing the positive role of the carbon nanofibers in the promotion of the H_2_ evolution activity. This increased activity mainly arises from the synergistic effect of the two composites, which results in evidently improved charge carrier transfer, an enlarged surface area, a decreased energy barrier and enhanced visible light absorption. It should be noted that the H_2_ production rate of the g-C_3_N_4_@C binary photocatalysts is 3.6 times higher than that of Pt/g-C_3_N_4_ (46.65 μ mol h^–1^), thus indicating the potential of noble-metal free carbon nanofibers for promoting H_2_ evolution. A remarkable AQY of 14.3% at 420 nm for the g-C_3_N_4_@C sample was achieved, which is much higher than that of pure g-C_3_N_4_ polymer photocatalysts, even in the presence of Pt-based cocatalysts.

Based on the above discussions, we can conclude that loading a small amount of appropriate cocatalyst on the surfaces of light excited photocatalysts could largely decrease the surface activation energy and improve the surface redox reaction rate. Therefore, it could largely promote the photocatalytic water reduction activity. However, the composition, morphology, properties and loading technique could largely affect the photocatalytic activities. In order to better compare the roles of different H_2_ evolution cocatalysts, we then analysed the photocatalytic performances of the cocatalysts. As is shown in Table 1, it is clear to see that different cocatalysts always exhibit very different photocatalytic performances. Noble metals, transition metals and even carbon-based materials could all be developed for use as H_2_ evolution cocatalysts. In most cases, Pt was utilized to evaluate the photocatalytic H_2_ evolution activities due to the better activity and facile fabrication process. Until now, the optimum photocatalytic H_2_ evolution activity (38.8%) was obtained when Pt was deposited on the melon-based g-C_3_N_4_ under visible light irradiation.^28^ Thus, for a theoretical study, it is reasonable to deposit Pt nanoparticles as the H_2_ evolution cocatalysts for the overall water splitting study.

**Table 1:** Photocatalytic H_2_ evolution activities of graphitic carbon nitride polymers modified with different cocatalysts

Entry	Sample	Cocatalyst	Electron donor	*λ* [nm]	AQY [%]	Ref.
1	g-C_3_N_4_	Ni(OH)_2_	TEOA	420	1.1	80
2	g-C_3_N_4_	β-Ni(OH)_2_	TEOA	402	1.48	81
3	g-C_3_N_4_	NiS	TEOA	440	1.9	88
4	mpg-C_3_N_4_	MoS_2_	Lactic acid	420	2.1	57
5	EB-g-C_3_N_4_	MoS_*x*_	TEOA	545	8.3	60
6	g-C_3_N_4_	Graphene + Pt	Methanol	400	2.6	48
7	g-C_3_N_4_	C nanofibers	TEOA	420	14.3	85
8	CNU-ATCN	Pt	TEOA	420	8.8	37
9	NS-g-C_3_N_4_	Pt	TEOA	420	9.6	45
10	CN-NS	Pt	TEOA	420	26.1	21
11	g-CN-1	Pt	TEOA	405	50.7	28
				420	38.8	28
11	g-CN-1	Pt	TEOA	420	38.8	

## 4. Water oxidation half reaction

Water oxidation is a multiple complex reaction, and involves 4-electron transfer, O–H bond breaking and O–O molecular bond formation. Therefore, this process is always constrained by its large energy consumption and sluggish reaction kinetics, which generate the current moderate activity.^69^ Furthermore, as water oxidation takes place at a high oxidation potential (theoretically this is 1.23 V), this always induces oxidative corrosion of the light transducer photocatalyst. Therefore, it is necessary to increase the selectivity of hole oxidation. In principle, cocatalyst modification could decrease the energy barrier and restrict charge recombination. This also calls for improvement of the O_2_ evolution selectivity, which contributes to maintaining the intrinsic catalytic properties of the photocatalysts.

**Fig. 10:**
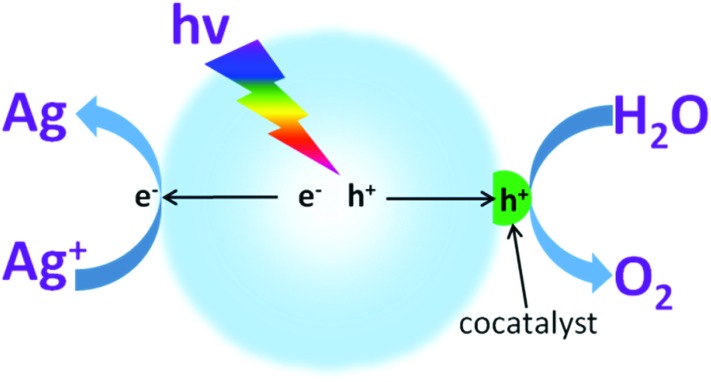
Schematic illustration of photocatalytic water oxidation for O_2_ evolution in the presence of an electron acceptor driven by a semiconductor modified with O_2_ evolution cocatalysts.

Normally, suitable cocatalysts that can dramatically decrease the activation energy and thus promote the water oxidation activity are highly desired (Fig. 10). Noble metals, such as RuO_2_ and IrO_2_, are known to be good catalysts for the water oxidation reaction.^65^ However, they always present high costs and toxicity, both of which greatly limit their sustainable applications for scaling up. Inspired by the plant cubene-like Mo_4_CaO_*x*_ cluster as the water oxidation active site in photosystem II, substantial efforts have been devoted to developing artificial water oxidation catalysts with sustainable components. Very recently, some first-row transition metal based materials *e.g.* CoPi,^97,98^ CoO_*x*_,^62,99,100^ and Co(OH)_2_ (ref. 101 and 102) have been reported to demonstrate comparable properties to those of Ru- and Ir-based noble metals for the water oxidation reaction, thus making them candidates for sustainable water oxidation development. For instance, when cobalt oxide nanoparticles were deposited on the surface of LaTiO_2_N, they exhibited much higher water oxidation activities than those of samples modified with IrO_2_.^103^ The AQY of the cobalt-modified LaTiO_2_N for O_2_ evolution at 440 nm reached as high as 27.1 ± 2.6%. This is mainly because the cobalt oxides can largely prolong the charge carrier lifetime. Consequently, more charge carriers are available for use in the subsequent photocatalytic water redox reactions. This certainly contributes to the improvement in the photocatalytic water oxidation activities. Importantly, compared with the Ru- and Ir-based noble metals, the cobalt based species possess many advantages, such as low-cost, low toxicity, abundant resources, versatile chemical states (Co^2+^ and Co^3+^) and excellent catalytic activities. Therefore, it is advised that cobalt based materials should be utilized as water oxidation cocatalysts in order to improve the photocatalytic water oxidation activities of g-C_3_N_4_ polymers.

In principle, pure g-C_3_N_4_ without any cocatalyst modification exhibit very low photocatalytic water oxidation activity even under strong UV light irradiation.^18,56^ This is mainly because the valence band potential of g-C_3_N_4_ is not positive enough to provide a sufficient driving force for the non-spontaneous water oxidation reaction. Furthermore, pure g-C_3_N_4_ is always defined by the fast charge carrier recombination rate, which decreases the water oxidation activity.^104^ In addition, the water oxidation process is hindered by the rather slow surface reaction kinetics, which are mainly attributed to the complex multielectron oxidation process and the huge activation energy barrier for O–O bond formation. In order to improve the photocatalytic water oxidation activities of the g-C_3_N_4_ polymers, it is advisable to deposit suitable cocatalysts on the surface of g-C_3_N_4_ to decrease the overpotential and accelerate the reaction kinetics.

As g-C_3_N_4_ has many lone-pair electrons, it can be used as an organic ligand to incorporate transition metals.^105^ Therefore, we firstly selected different transition metal ions (*e.g.* Fe^3+^, Ni^2+^ and Co^2+^) to incorporate with g-C_3_N_4_, with the aiming of fabricating an efficient water oxidation system containing sustainable elements.^106^ It is exciting to observe that cobalt modification could indeed improve the water oxidation activity of g-C_3_N_4_, thus indicating the positive role of cobalt in improving the water oxidation kinetics. However, the water oxidation activity of the system is closely related to the composition, structure, and properties of the cocatalysts, which are usually affected by the preparation strategy. Therefore, the cocatalyst modification technique should be optimized to further improve the water oxidation activities of the g-C_3_N_4_ polymers.

**Fig. 11:**
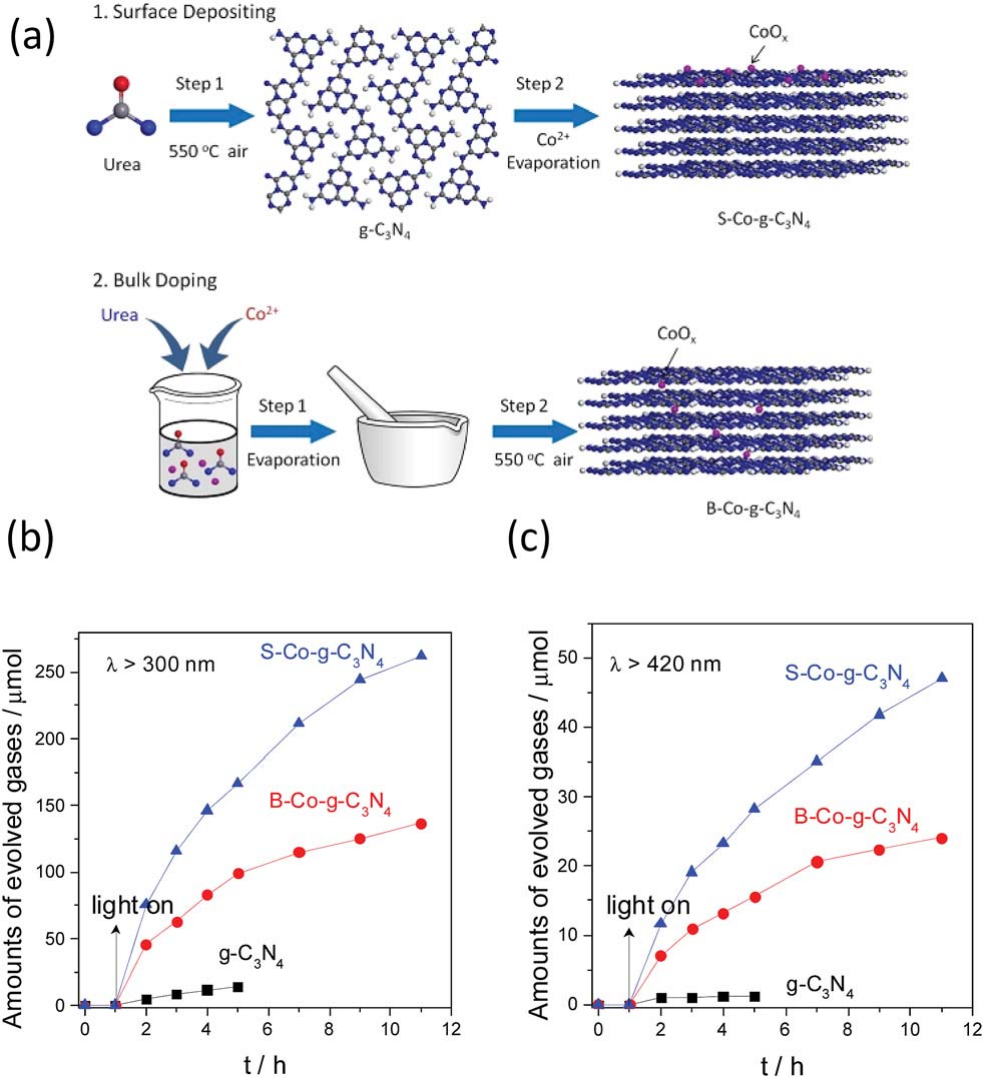
(a) Schematic illustration of surface modification and bulk doping modification of CoO_*x*_ with g-C_3_N_4_. Photocatalytic water oxidation activities of the g-C_3_N_4_, B–Co–g-C_3_N_4_ and S–Co–g-C_3_N_4_ samples under (b) UV (*λ* > 300 nm) and (c) visible light (*λ* > 420 nm) irradiation. Reprinted with permission from ref. 107. Copyright 2016, American Chemical Society.

We then investigated the effect arising from cocatalyst modification. As is shown in Fig. 11a, two different modification techniques, based on bulk doping and surface deposition, have been developed to study the property–activity relationship.^107^ It should be noted that surface deposition with cobalt demonstrated great advantages in terms of improving the water oxidation activity in comparison with the bulk doping modification. As is shown in Fig. 11b, pure g-C_3_N_4_ exhibited low activity towards O_2_ evolution (5 μmol h^–1^) under UV light irradiation. When cobalt was doped into the framework of g-C_3_N_4_, the O_2_ evolution rate increased up to a value of 46 μmol h^–1^, which is 9 times higher than that of pure g-C_3_N_4_. The O_2_ evolution activity was further enhanced to 75.6 μmol h^–1^ when the same amounts of cobalt were deposited on the surface of g-C_3_N_4_. This is not difficult to understand, because more active sites would be exposed on the surfaces of the polymers, which is believed to maximize the activity. On the contrary, most of the active sites would be embedded into the bulk of the polymer framework, which decreases the interface water oxidation activity. The same enhanced water oxidation activities were also obtained when the samples were examined with visible light (Fig. 11c), thus elucidating well the huge advantage of surface deposition modification.

Except for the cobalt oxides, other cobalt based materials deposited on the surface of g-C_3_N_4_ could also decrease the energy barrier and accelerate the water oxidation reaction rate. For instance, when layered Co(OH)_2_ is deposited on the surface of g-C_3_N_4_ nanosheet,^102^ it will creates an intense adhesion between light transducer and water oxidation cocatalyst due to the similar 2D layered geometry, which to some extent will favour the interfacial charge carrier transfer and promote the water oxidation activities. Indeed, Co(OH)_2_ modified g-C_3_N_4_ showed obviously increased O_2_ evolution activities in comparison with the pristine g-C_3_N_4_. After 4 hours of persistent reaction under visible light irradiation, the total amounts of the evolved O_2_ gases could be reached 14 μmol for Co(OH)_2_ modified g-C_3_N_4_, while only 1.3 μmol of O_2_ gases were examined for pure g-C_3_N_4_. Other metal hydroxides, *i.e.* Fe(OH)_3_, Ni(0H)_2_, and Cu(OH)_2_ have also been deposited on the interface of g-C_3_N_4_ for water oxidation study, however, they only showed slightly or no increase in the water oxidation activities, demonstrating the advantage of cobalt based materials in promoting the water oxidation activities.

In addition, TMDs-based cobalt species, such as CoS_2_ and CoSe_2_, have also been developed to act as excellent water oxidation cocatalysts for improving the O_2_ evolution rate.^108,109^ By virtue of the different kinds of the cobalt species, they always exhibited different behaviours in promoting the water oxidation activities. Till now, the highest AQY of 1.1% at 420 nm for visible light driven water oxidation have been obtained on Co_3_O_4_ nanoparticles modified sulfur-mediated g-C_3_N_4_ polymers.^62^ Note that pristine g-C_3_N_4_ was barely active for water oxidation even under intensive UV irradiation. It is thus of pivotal interest to observe that cobalt based materials could indeed advance the interface charge carrier mobility and optimize the photocatalytic water oxidation performance.

Based on the above discussions, we can conclude that the cobalt based materials are excellent cocatalysts to promote the photocatalytic water oxidation activities of the g-C_3_N_4_ polymers in terms of largely decreasing the huge energy barrier and facilitating the sluggish reaction kinetics. Therefore, with the major purpose of achieving the 4-electron overall water splitting process, cobalt based materials are certainly regarded as the promising O_2_ evolution cocatalysts to improve the water oxidation half reaction.

## 5. Overall water splitting

Based on the above experimental observations, we can conclude that Pt and cobalt based materials are the most favourable H_2_ and O_2_ evolution cocatalysts, respectively, to separately promote the water reduction and water oxidation half reactions of g-C_3_N_4_ polymers. It is expected that overall water splitting should be achieved when the optimal H_2_ and O_2_ evolution cocatalysts are simultaneously deposited on the surfaces of g-C_3_N_4_ polymers to separately promote the water reduction and oxidation half reactions, respectively. Following this hypothesis, we intend to control the surface reaction kinetics of the g-C_3_N_4_ polymers through rational modification of the H_2_ and O_2_ evolution cocatalysts.

Traditionally, the catalytic activities of cocatalysts are highly dependant on their physicochemical properties, such as particle size, morphology and structure.^65^ Therefore, the loading technique may be of pivotal significance for achieving photocatalytic overall water splitting. Three traditional reduction techniques, based on NaBH_4_ reduction, H_2_ reduction and *in situ* photo-reduction, were developed to deposit Pt-based species on the surface of g-C_3_N_4_ for a water splitting study.^110^ Excitingly, it was found that, different from the water reduction activity in the presence of sacrificial agents, only when Pt species are homogeneously loaded on the g-C_3_N_4_ polymers by *in situ* photo-reduction can overall water splitting with persistent H_2_ and O_2_ evolution be realized. Otherwise, only very small amounts of H_2_ evolution and nearly no O_2_ evolution were observed. High resolution XPS analysis revealed that both Pt and PtO_*x*_ were generated by *in situ* photo-deposition, whereas only Pt was generated for the H_2_ and NaBH_4_ reduction, prepared using an immersion strategy. It was previously found that oxidized platinum could to some extent promote the water oxidation process. Therefore, both H_2_ and O_2_ evolution cocatalysts could be generated by *in situ* photodeposition, which may play a key role in triggering overall water splitting, otherwise only H_2_ evolution cocatalysts could be created. Under these circumstances, without the assistance of O_2_ evolution cocatalysts, the hole oxidation process is rather slow and the water oxidation process is extremely impeded, which would therefore increase the charge carrier recombination rate and generally decrease the overall water splitting activity. Moreover, in the absence of water oxidation cocatalysts, the water splitting reverse reaction, namely spontaneous water formation, could also immediately occur, which may also further decrease the overall water splitting reaction.

**Fig. 12:**
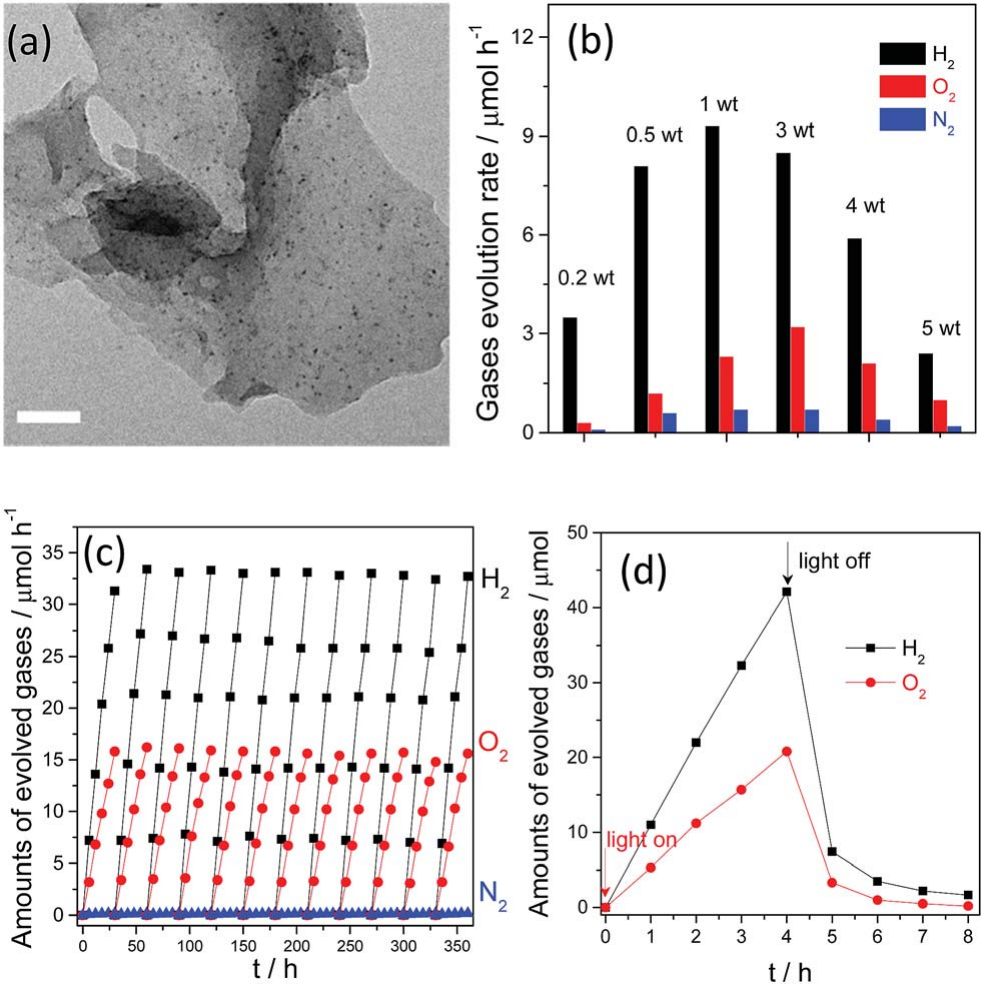
(a) TEM image of Pt deposited g-C_3_N_4_ polymers prepared by *in situ* photo-reduction; (b) overall water splitting activities of g-C_3_N_4_ polymers deposited with different amounts of Pt; (c) long reaction time overall water splitting by a Pt–Co–g-C_3_N_4_ polymer; (d) overall water splitting activities with the light on and off. Reprinted with permission from ref. 110. Copyright 2016, Royal Society of Chemistry.

Therefore, the simultaneous formation of dual Pt and PtO_*x*_ as H_2_ and O_2_ evolution cocatalysts by *in situ* photodeposition is believed to be a requirement for overall water splitting, as they can be selectively deposited on the active sites of g-C_3_N_4_ polymers as kinetic promoters in order to promote the water splitting reaction. HR-TEM images (Fig. 12a) further reveal that ultrafine Pt nanoparticles with average sizes of about 2–3 nm were homogeneously generated by *in situ* photo-deposition, and were believed to exhibit excellent activities for catalytic reactions. Conversely, large sized Pt particles were obtained when Pt was prepared by rough reduction from H_2_ or NaBH_4_ solution. Evidently, the large particle sizes of the catalysts usually decrease the number of available active sites and have proven to be insufficient for improving the water splitting activity.

Furthermore, the molecular ratio of the evolved H_2_ and O_2_ gases, which in fact represents the water reduction and oxidation half reaction rates, could be finely tuned by simply varying the initial loading amounts of the platinum precursors. As is shown in Fig. 12b, the molecular ratio of H_2_ and O_2_ evolution clearly changed when the Pt and PtO_*x*_ loading amounts were varied from 0.2 wt% to 5 wt%. When the loading content was increased from 0.2 wt% to 1 wt%, both the H_2_ and O_2_ evolution rates increased. Further increasing the loading amount decreased the H_2_ evolution rate, while the optimal O_2_ evolution rate was observed when the cocatalyst amount was confirmed as 3 wt%. It can also be seen that the molecular ratio of H_2_ and O_2_ evolution is slightly lower than 2 : 1, and is in close proximity to the stoichiometric ratio of overall water splitting. However, accompanying H_2_ and O_2_ evolution, evident N_2_ evolution was observed, which is probably due to self-oxidation of the g-C_3_N_4_ polymers by the valence band holes, and this would certainly decrease the O_2_ evolution selectivity and activity. This self-oxidation of the photocatalyst indeed promotes catalyst corrosion and is harmful to the overall efficiency from the viewpoint of the atom economy, which is mainly due to the absence of efficient water oxidation cocatalysts. Despite the fact that PtO_*x*_ can promote the water oxidation reaction, it only demonstrates low efficiency and low selectivity for O_2_ evolution. Thus, it should be expected that the photocatalytic overall water splitting performance, especially the O_2_ evolution selectivity, could be further optimized when cobalt oxides are deposited as water oxidation cocatalysts. Actually, nearly no N_2_ evolution was obtained when a small amount (1 wt%) of CoO_*x*_ was co-loaded as a water oxidation cocatalyst (Fig. 12c). A similar optimization was also observed when CoP was used as the water oxidation cocatalyst for overall water splitting.^111^ Meanwhile, the simultaneous loading of Pt and Co as excellent H_2_ and O_2_ evolution cocatalysts would greatly benefit the stability of the g-C_3_N_4_ polymers. After 500 hours of persistent reaction, nearly no evident decrease in the activity was observed for the Pt–Co–g-C_3_N_4_ photocatalyst, thus indicating its robust stability toward solution corrosion. Finally, overall water splitting with a stoichiometric ratio of 2 : 1 for H_2_ and O_2_ evolution could be achieved after *in situ* modification with Pt, PtO_*x*_ and CoO_*x*_ as the H_2_ and O_2_ evolution promoters. The currently achieved AQY of the system under visible light irradiation is only 0.3%. The relatively low efficiency is probably hindered by the fast and spontaneous reverse reaction, namely water formation (Fig. 12d). This is because although noble metals such as Pt and Rh are excellent promoters for H_2_ evolution, they can also function as good catalysts to promote the water formation reaction, which is thermodynamically spontaneous and is much easier to achieve than the non-spontaneous water splitting reaction. The evolved H_2_ and O_2_ gases immediately react on the surface of the cocatalyst to drive the water formation reaction. Therefore, further investigations based on surface nanostructure engineering of both cocatalysts and the photocatalysts to prevent the reverse reaction are believed to be an efficient route to improving the overall water splitting activity.

**Fig. 13:**
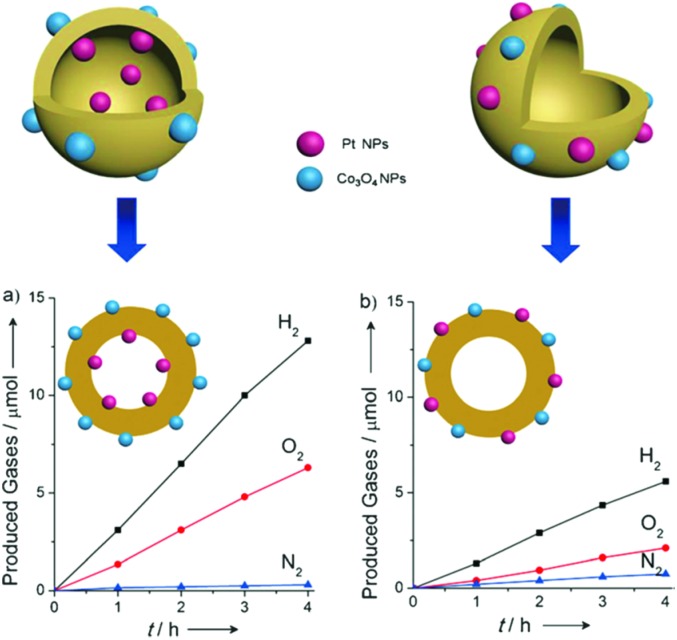
Time course of the photocatalytic evolution of H_2_ and O_2_ using (a) Co_3_O_4_/HCNS/Pt and (b) (Co_3_O_4_ + Pt)/HCNS under UV irradiation (*λ* > 300 nm). Reprinted with permission from ref. 40. Copyright 2016, Wiley-CVH.

A prototypical example of this is a hollow sphere carbon nitride (HSCN) with a Janus interface to individually deposit Pt and Co_3_O_4_ nanoparticles on the inside and outside interfaces, thus spatially separating the H_2_ and O_2_ evolution active sites and so avoiding the reverse reaction.^40^ As is shown in Fig. 13, when the Pt and Co_3_O_4_ nanoparticles were separately deposited on the inside and outside interfaces of HSCN, the photocatalytic H_2_ and O_2_ evolution activity was much higher than that with both of the cocatalysts loaded on the outside interface, thus reflecting the fact that the reverse reaction was indeed restricted.

## 6. Conclusions and outlook

Overall water splitting for the stoichiometric generation of H_2_ and O_2_ has been achieved by rational cocatalyst modification of g-C_3_N_4_ polymers to modulate the surface redox reaction kinetics. It was found that Pt and CoO_*x*_ are excellent H_2_ and O_2_ evolution cocatalysts that decrease the activation energy barrier and accelerate the reaction kinetics of the g-C_3_N_4_ polymers. The current apparent quantum yield (AQY) for overall water splitting is calculated to be 0.3% at 405 nm. It should be noted that the reverse reaction, namely water formation, is also found to accompany the water splitting reaction. Furthermore, the competing reverse reaction is a thermodynamically downhill reaction; it can occur spontaneously and largely decreases the overall water splitting activity. Hence, future research should be directed towards restricting the reverse reaction, with the aim of further improving the overall water splitting activity. In addition, by virtue of the wide variety of conjugated polymers and their versatile structural variations from solution based organic protocols, future development of sustainable water splitting systems composed of new organic conjugated polymers are therefore envisaged. Furthermore, a careful and in depth understanding of the property–activity relationships between cocatalysts and carbon nitride would also provide promising new insight which will aid in the design of efficient systems for other catalytic reactions, such as CO_2_ reduction. Such a research topic is indeed very interesting, and some fascinating results have recently been achieved by scientists.^112–116^
